# Functional Diversity of TonB-Like Proteins in the Heterocyst-Forming Cyanobacterium *Anabaena* sp. PCC 7120

**DOI:** 10.1128/mSphere.00214-21

**Published:** 2021-11-17

**Authors:** Hannah Schätzle, Sergio Arévalo, Leonard Fresenborg, Hans-Michael Seitz, Enrique Flores, Enrico Schleiff

**Affiliations:** a Institute for Molecular Biosciences, Goethe University Frankfurt, Frankfurt am Main, Germany; b Frankfurt Isotope and Element Research Center (FIERCE), Goethe University Frankfurt, Frankfurt am Main, Germany; c Buchmann Institute for Molecular Life Sciences, Frankfurt am Main, Germany; d Instituto de Bioquímica Vegetal y Fotosíntesis, CSIC and Universidad de Sevilla, Seville, Spain; e Frankfurt Institute for Advanced Studies, Frankfurt am Main, Germany; f Institute for Geoscience, Petrology and Geochemistry, Goethe University Frankfurt, Frankfurt am Main, Germany; University of Minnesota

**Keywords:** *Anabaena*, TonB protein, cyanobacteria, metal transport, nitrogenase, outer membrane, siderophores

## Abstract

The TonB-dependent transport of scarcely available substrates across the outer membrane is a conserved feature in Gram-negative bacteria. The plasma membrane-embedded TonB-ExbB-ExbD accomplishes complex functions as an energy transducer by physically interacting with TonB-dependent outer membrane transporters (TBDTs). TonB mediates structural rearrangements in the substrate-loaded TBDTs that are required for substrate translocation into the periplasm. In the model heterocyst-forming cyanobacterium *Anabaena* sp. strain PCC 7120, four TonB-like proteins have been identified. Out of these TonB3 accomplishes the transport of ferric schizokinen, the siderophore which is secreted by *Anabaena* to scavenge iron. In contrast, TonB1 (SjdR) is exceptionally short and not involved in schizokinen transport. The proposed function of SjdR in peptidoglycan structuring eliminates the protein from the list of TonB proteins in *Anabaena*. Compared with the well-characterized properties of SjdR and TonB3, the functions of TonB2 and TonB4 are yet unknown. Here, we examined *tonB2* and *tonB4* mutants for siderophore transport capacities and other specific phenotypic features. Both mutants were not or only slightly affected in schizokinen transport, whereas they showed decreased nitrogenase activity in apparently normal heterocysts. Moreover, the cellular metal concentrations and pigment contents were altered in the mutants, most pronouncedly in the *tonB2* mutant. This strain showed an altered susceptibility toward antibiotics and SDS and formed cell aggregates when grown in liquid culture, a phenotype associated with an elevated lipopolysaccharide (LPS) production. Thus, the TonB-like proteins in *Anabaena* appear to take over distinct functions, and the mutation of TonB2 strongly influences outer membrane integrity.

**IMPORTANCE** The genomes of many organisms encode more than one TonB protein, and their number does not necessarily correlate with that of TonB-dependent outer membrane transporters. Consequently, specific as well as redundant functions of the different TonB proteins have been identified. In addition to a role in uptake of scarcely available nutrients, including iron complexes, TonB proteins are related to virulence, flagellum assembly, pilus localization, or envelope integrity, including antibiotic resistance. The knowledge about the function of TonB proteins in cyanobacteria is limited. Here, we compare the four TonB proteins of *Anabaena* sp. strain PCC 7120, providing evidence that their functions are in part distinct, since mutants of these proteins exhibit specific features but also show some common impairments.

## INTRODUCTION

Cyanobacteria possess a Gram-negative type of cell envelope containing an outer membrane (OM), a peptidoglycan (PG) layer, and a plasma (cytoplasmic or inner) membrane (PM) (1). Macromolecular complexes that reside in the two membranes facilitate the assembly of the cell wall components as well as solute exchange and signaling (2). Among them, the OM-embedded TonB-dependent transport machinery is widely distributed in Gram-negative bacteria (3). The TonB-dependent transport system is important for growth under iron starvation conditions, since iron is an essential but scantily bioavailable nutrient (3–7). Iron-loaded proteins carry out functions in important cellular activities such as electron transport and DNA synthesis (8). This holds particularly true for cyanobacteria, in which iron is required for the synthesis of phycobiliproteins (9) and chlorophyll *a* (Chl) (10), as well as for photosynthetic complexes that in total require approximately 22 to 23 iron atoms (11). Moreover, in certain cyanobacterial species that are capable of nitrogen fixation, the nitrogenase enzyme is also dependent on iron (12).

Due to its low solubility under oxic conditions at neutral pH, iron rapidly forms insoluble aggregates that are inaccessible to many microorganisms (6, 13). Only a very small amount of dissolved iron exists as inorganic iron, whereas the largest proportion is bound to organic ligands such as siderophores (6). Siderophores are low-molecular-weight compounds that chelate ferric iron with high affinity. The production and secretion of siderophores is a widespread strategy of bacteria, fungi, and plants to cope with iron-limiting conditions (14). Siderophores are divided into three classes depending on the chemical nature of iron coordination, namely, catecholates, hydroxamates, or mixed types that contain another iron complexing group such as hydroxycarboxylate (15).

The TonB-dependent transport system involves a PM-localized energizing TonB-ExbB-ExbD complex and OM-localized TonB-dependent transporters (TBDTs). TBDTs constitute gated channels that facilitate the transport of substrates into the periplasm (16). The translocation process is energy dependent, as the substrates are typically large and rarely abundant (17, 18). Examples besides siderophores are carbohydrates, vitamin B_12_ (cobalamin), and heme (16, 19). The energy for transport is derived from the proton motive force (pmf) across the PM (20, 21). ExbB and ExbD build up a proton channel that converts the pmf into energy for the translocation process (22). The TonB protein transfers the energy to the TBDT through direct interaction with both, ExbB/ExbD and the TBDT (23–26).

TonB proteins contain a transmembrane α-helix and a conserved C-terminal motif that interacts with the so-called TonB box of the TBDTs (16, 27). Remarkably, more than 40% of the organisms that possess a TonB-dependent system have more than one *tonB* gene copy (3). For instance, Pseudomonas aeruginosa possesses three TonB proteins (28–30). Here, TonB1 and likely TonB2 facilitate the transport of iron-containing compounds and are required for growth under iron-limiting conditions, while TonB3 is crucial for motility and pilus assembly (28–32). In Pseudomonas putida, one of the two TonB proteins energizes the transport of siderophores, whereas the other TonB protein is important for maintaining the integrity of the cell envelope and flagellum localization (33–35). Also, *Vibrio* species typically contain multiple *tonB* copies in the genome. Here, distinct TonB proteins facilitate the transport of both common and individual substrates (36). Thus, multiple TonB proteins in one organism can take over redundant as well as unique functions. They can function in protein complex assembly, cell wall integrity regulation, or global or substrate-specific transport processes.

Little is known about the functionality of TonB proteins in cyanobacteria, which are photoautotrophic organisms that can be found in terrestrial, marine, or freshwater habitats. The number of putative TBDT, TonB, or ExbB/D proteins in the genomes of analyzed cyanobacteria is highly variable (37, 38). For example, in the genome of the filamentous cyanobacterium *Anabaena* sp. strain PCC 7120 (*Anabaena* hereafter) 22 different TBDTs were predicted (37). In contrast, only four genes with a *tonB* signature were assigned by bioinformatics methods (38). TonB1 contains an exceptionally short periplasmic domain that is likely not sufficient in size to reach OM-embedded factors. In contrast, TonB3 is supposed to be a central component of the ferric siderophore transport system (38). The *tonB3* mRNA abundance increases under iron-limited conditions, and a single recombination mutant can be generated only in the presence of enhanced iron concentrations (38). The growth of this mutant in the absence of iron is reduced, and siderophore synthesis genes are upregulated in this genetic background (38). Further, we could show recently that the transport of ferric schizokinen, the siderophore secreted by *Anabaena*, was abolished in a *tonB3* mutant (39). In contrast, the *tonB1* mutant retained the siderophore transport capacity but was severely impacted in diazotrophic growth. This could be traced back to an abnormal peptidoglycan morphology in the heterocyst septa of the mutant, and therefore, TonB1 was renamed septal junction disc regulator (SjdR) (39).

**FIG 1:**
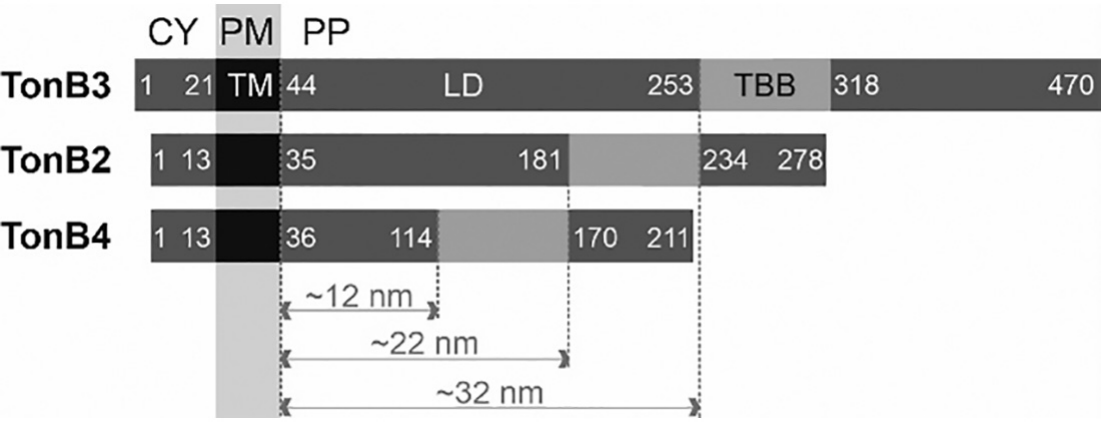
Domain architecture of TonB proteins in *Anabaena*. TonB3, TonB2, and TonB4 contain an N-terminal cytosolic region (CY), a transmembrane region (TM), a linker domain (LD), the TonB-box binding region (TBB), and a C-terminal extension. Indicated are the amino acids at the border of the cytosolic region, the LD, and the C-terminal extension. The length of the linker domain was calculated assuming an extended helix (3.6 amino acids and 0.54 nm per turn). PM, plasma membrane; PP, periplasm.

TonB2 encoded by *all3585* or TonB4 encoded by *alr5329* has a domain structure comparable to the TonB proteins from Escherichia coli or TonB3 from *Anabaena* (38). However, the distance between the transmembrane domain and the TonB-box binding domain is smaller than in TonB3 (Fig. 1). Estimation of the size assuming an extended helix suggests a dimension of 12 nm for TonB4 and 22 nm for TonB2, while for TonB3 a 32-nm size is estimated. The latter fits the determined distance between OM and PM in *Anabaena*, as well as the estimated size of the TolC system (40, 41). Both *tonB2* and *tonB4* are expressed at highest levels in low-density cultures and lowest levels in the stationary phase (42). Their expression is enhanced at all growth stages in the presence of elevated iron (38, 42), while the expression of *tonB4* is also enhanced in the presence of elevated copper concentrations (38, 42).

Considering the essential role of TonB3 in siderophore transport, opposed to the novel functionality of SjdR, which is not related to TonB-dependent transport, we now aimed to characterize the TonB-like proteins TonB2 and TonB4, since the role of those proteins in *Anabaena* is still unclear (38, 39). Insertion mutants for *tonB2*, *tonB3*, and *tonB4* demonstrated alterations in cellular metal levels as well as in carotenoid (Car) or chlorophyll *a* concentrations compared to the wild type, although to different extents. Moreover, the *tonB2* mutant filaments aggregated in liquid cultures, which might be related to an enhanced production of lipopolysaccharide in this strain. Also, the outer membrane integrity as well as the expression of porins was affected in the *tonB2* mutant. On the other hand, the *tonB2* mutant as well as the *tonB4* mutant retains the siderophore transport capacity, which suggests a functional diversity of *Anabaena* TonB proteins.

## RESULTS

### The *Anabaena tonB* mutants bear pigment alterations.

**FIG 2:**
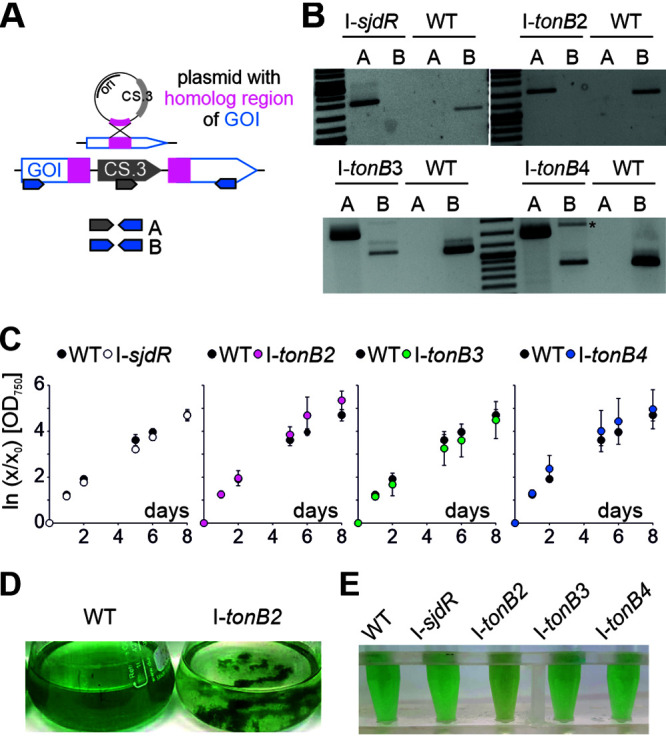
Growth phenotype of the *tonB* mutants and the *sjdR* mutant strains. (A) Illustration of the single recombination strategy; a portion of the gene of interest (GOI) is cloned into a plasmid which bears the Sp^r^ Sm^r^ cassette (CS.3). After the recombination event the GOI in the mutant is interrupted by plasmid insertion. The oligonucleotide pairs utilized for mutant screening are indicated; primer combination A results in a product when the plasmid is integrated into the genome; combination B can generate a product only when the GOI is intact. (B) Segregation analyses of I-*tonB3* and I-*sjdR* were described previously (38, 39). Either an oligonucleotide pair specific for the insertion fragment was used (lanes A), or an oligonucleotide pair specific for the wild-type gene (lanes B). The asterisk marks an unspecific PCR product. (C) The growth of the indicated strains in YBG11 medium was determined by analysis of the OD_750_; the wild-type experiment is shown in the four diagrams for better comparability to the behavior of each mutant. Values were normalized to initial OD_750_ of the culture and are expressed as natural logarithm. The mean from at least three biological replicates is shown with the standard deviation as error bar. (D) Wild-type and I-*tonB2* cultures photographed after growth for 7 days in YBG11 medium. (E) Wild-type and mutant cultures photographed after growth for 5 days in YBG11 medium; the I-*tonB2* sample was homogenized by pipetting up and down prior to the photographing in order to reduce clumping.

To analyze putative functions of the individual TonB proteins, the growth behavior of mutants of the corresponding genes was examined. The mutant strains, I-*sjdR*, I-*tonB2*, I-*tonB3*, and I-*tonB4*, were generated through single recombination insertion of a plasmid in the gene of interest (Fig. 2A), as described before (38, 39). In accordance with previous reports, I-*sjdR* and I-*tonB2* were segregated, as no wild-type copy of the respective genes was detectable in the mutants (Fig. 2B) (39). In contrast, I-*tonB3* and I-*tonB4* could not be segregated, as even after repeated dilution on plates with antibiotics, the wild-type genes were detectable in the corresponding genomic DNA (gDNA) by PCR (Fig. 2A) (38). For I-*tonB3* this is consistent with the previous report, where full segregation was obtained only in the presence of enhanced iron (38). This suggests that TonB3 and TonB4 are important for viability under the conditions used in this study.

None of the *tonB* mutants exhibited an altered growth behavior compared to wild type under standard conditions (YBG11 medium, Fig. 2C). However, I-*tonB2* cells frequently formed aggregates in liquid medium (Fig. 2D). The enhanced tendency of I*-tonB2* to aggregate was also verified by sedimentation analysis (see Fig. S1 in the supplemental material). In addition, the color of I-*tonB2* was considerably different from that of the wild type (Fig. 2E), suggesting a modification in the cellular pigment content.

10.1128/mSphere.00214-21.1 FIG S1 Sedimentation of I-*tonB2* (white symbols) and wild-type cultures (black symbols). The OD_750_ was measured in one cuvette after specific time points without further resuspension of the suspension. Average values of four biological replicates per strain with two technical replicates each are given; error bars show the standard deviation. Due to enhanced aggregation, I-*tonB2* filaments sediment faster in the cuvette than wild-type samples, which is indicated by a faster decrease of the OD_750_. Download FIG S1, PDF file, 1.4 MB. Copyright © 2021 Schätzle et al. 2021 Schätzle et al. https://creativecommons.org/licenses/by/4.0/ This content is distributed under the terms of the Creative Commons Attribution 4.0 International license.

The synthesis of carotenoids and Chl in *Anabaena* is differentially regulated in response to growth temperature and light intensity (43–46). Therefore, the concentrations of these pigments in the mutants and the wild type were determined after growth of the cultures for 7 days under ambient light (70 μmol photons m^−2^ s^−1^) as well as under high-light or low-light conditions (140 and 15 μmol photons m^−2^ s^−1^, respectively).

In general, the pigment concentrations under ambient light and low light were comparable. When grown under ambient or low-light conditions, the Chl concentration in the wild type was 9 ± 2 μg ml^−1^ at an optical density at 750 nm (OD_750_) of 1, the carotenoid concentration was 2.5 ± 0.3 μg ml^−1^ at an OD_750_ of 1, and the phycocyanin (PC) concentration was 26 ± 7 μg ml^−1^. Compared to that, all mutant strains were diminished in their cellular Chl content under both conditions (Table 1) even though a significant difference was found only for I-*sjdR* (low light) and I-*tonB4* (ambient and low light). TonB3 is involved in siderophore transport (38, 39), and because a reduction in Chl is an indicator of iron starvation in *Anabaena*, the decrease in Chl possibly mirrors a fast iron starvation (47, 48). SjdR, however, is not involved in TonB-dependent schizokinen transport (39), and therefore, the observed reduction of the Chl content should not be related to iron uptake. Likewise, TonB4 is not involved in iron uptake, and the cause of Chl decrease remains elusive.

**TABLE 1:** Chlorophyll *a*, carotenoid, and phycocyanin concentrations in the wild type and the *tonB* mutants^*a*^

Strain	High light				Ambient light				Low light			
	Chl/OD_750_ (μg ml^−1^)	Car/OD_750_ (μg ml^−1^)	Ratio, Chl/Car	PC/OD_750_ (μg ml^−1^)	Chl/OD_750_ (μg ml^−1^)	Car/OD_750_ (μg ml^−1^)	Ratio, Chl/Car	PC/OD_750_ (μg ml^−1^)	Chl/OD_750_ (μg ml^−1^)	Car/OD_750_ (μg ml^−1^)	Ratio, Chl/Car	PC/OD_750_ (μg ml^−1^)
WT	7 ± 2	2.2 ± 0.2	3 ± 1	27 ± 3	9 ± 2	2.4 ± 0.3	3.6 ± 0.3	27 ± 6	9 ± 1	2.5 ± 0.4	3.7 ± 0.3	25 ± 3
I-*sjdR*	5 ± 2	**1.6 ± 0.4**	3 ± 1	22 ± 5	7 ± 2	2.0 ± 0.7	3.6 ± 0.5	21 ± 7	**7 ± 2**	**1.8 ± 0.6**	3.9 ± 0.8	24 ± 5
I-*tonB2*	4.4 ± 0.8	2 ± 1	2 ± 1	21 ± 3	7 ± 3	2 ± 1	3.0 ± 0.6	**12 ± 2**	7 ± 3	3 ± 1	3.2 ± 0.8	**15 ± 4**
I-*tonB3*	6 ± 2	2.1 ± 0.5	2.7 ± 0.9	30 ± 3	7 ± 2	2.6 ± 0.8	**2.9 ± 0.5**	24 ± 7	8 ± 2	2.3 ± 0.4	3.4 ± 0.9	28 ± 5
I-*tonB4*	5 ± 2	2.0 ± 0.3	3 ± 1	27 ± 9	**7 ± 1**	**2.0 ± 0.4**	3.6 ± 0.5	24 ± 9	**7 ± 1**	**1.8 ± 0.5**	4.2 ± 0.5	21 ± 6

a Given are the concentrations in cultures grown for 7 days under high, ambient, or low light (140, 70, or 15 μmol photons m^−2^ s^−1^, respectively) in YBG11 medium. The average from 4 to 10 biological replicates and the standard deviation are given normalized to an OD_750_ of 1. Values in the mutants that significantly differ from wild-type values are indicated in bold (*P* < 0.05, Student’s *t* test with Bonferroni correction). Chl, chlorophyll *a*; Car, carotenoid; PC, phycocyanin.

Notably, I-*tonB3*, with 2.6 ± 0.8 μg ml^−1^, exhibited an elevated carotenoid concentration under ambient light compared to wild type. Under low light, the I-*tonB2* carotenoid concentration was enhanced. In contrast to that, I-*tonB4* had a significantly lower carotenoid level under both ambient and low-light conditions, and I-*sjdR* under low light. There were no significant differences of chlorophyll-to-carotenoid ratio under ambient or low-light conditions with the exception of a small decrease in I-*tonB3* (ambient light). Under both conditions, I-*tonB2* and I-*tonB3* had lower ratios than the wild type and the other mutants (Table 1).

The concentrations of phycocyanin were similar in all strains except I-*tonB2*, in which it was significantly decreased by a factor of 1.5 and 2 (low and ambient light, respectively).

The Chl concentration was higher in all strains under normal (ambient) light or low-light conditions compared to high light (Table 1). For the wild-type strain, an average Chl concentration per OD_750_ of 7 ± 1 μg ml^−1^ was determined under high light. Overall, the carotenoid level tended to be lower under high light than under ambient light as well (Table 1), but the difference between these conditions was not as drastic as in the case of Chl. This resulted in a lowered ratio of Chl to Car. These data are consistent with previous observations (49–51). Only in I-*sjdR* was the carotenoid content significantly decreased compared to the wild type (Table 1). This reduction did not result in compromised growth (Fig. 1). Phycocyanin content did not differ significantly between the strains under high light. While the PC content of I-*tonB2* increased relative to ambient light, the PC content of the other strains did not differ significantly between light conditions.

In summary, I-*tonB2* contained a strongly lowered level of phycocyanin under ambient and low-light conditions and all mutants showed a mildly lower chlorophyll content compared to wild type under the same condition. Therefore, the color alterations observed for I-*tonB2* likely result from the observed alterations in cellular pigmentation.

### The cellular metal content is altered in *tonB* mutants.

The carotenoid concentration in cyanobacteria is affected by metal availability. Elevated Cu, Zn, or Co concentrations result in an elevation of the carotenoid content in Anabaena oryzae (52). Similarly, Ca supplementation enhances the level of pigments in *Anabaena* (53, 54). Thus, the cellular metal concentrations in I-*tonB2*, I-*tonB3*, and I-*tonB4* were determined by inductively coupled plasma mass spectrometry (ICP-MS) analyses and compared to the wild-type concentrations that were described before (39) Since SjdR is functionally not related to TonB-dependent transport (39), this strain was excluded from the further studies.

Remarkably, alterations in metal concentrations were observed for all *tonB* mutants compared to the wild type. (i) I-*tonB3* and I-*tonB4* exhibited a decrease in cellular Mg and Co concentrations compared to the wild type (Table 2). (ii) In I-*tonB2* and I-*tonB3* the Mn concentration was decreased. In I-*tonB4* the level of Mn showed a large variation, but Mn was always at a lower level than in the wild type. The Mo concentration was (iii) enhanced in I-*tonB2* and (iv) reduced in I-*tonB4*. (v) In I-*tonB2* cells an elevated Cu concentration was observed compared to the wild type. Notably, after 7 days of growth in YBG11 medium an alteration in the Fe concentration was not observed, although TonB3 is supposed to be involved in ferric siderophore transport.

**TABLE 2:** Metal concentration in wild-type *Anabaena* and the mutants I-*tonB2*, I-*tonB3*, and I-*tonB4* expressed as atoms per OD_750_^*a*^

Metal	10^13^ atoms/OD				Ratio (mutant/WT)		
	WT	I-*tonB2*	I-*tonB3*	I-*tonB4*	I-*tonB2*	I-*tonB3*	I-*tonB4*
Mg	500 ± 40	410 ± 20	350 ± 40	280 ± 10	0.82	**0.70**	**0.56**
Ca	61 ± 7	70 ± 50	60 ± 20	40 ± 20	1.15	0.98	0.66
Mn	51 ± 2	17 ± 1	11 ± 1	30 ± 20	**0.33**	**0.22**	0.59
Fe	34 ± 2	35 ± 5	29 ± 3	30 ± 8	1.03	0.85	0.88
Co	1.8 ± 0.1	1.8 ± 0.1	1.2 ± 0.1	1.3 ± 0.1	1.00	**0.66**	**0.72**
Cu	5.1 ± 0.5	6.7 ± 0.6	5 ± 2	6 ± 1	**1.31**	0.98	1.18
Zn	5.4 ± 0.3	6.1 ± 0.4	5 ± 4	7 ± 3	1.13	0.93	1.29
Mo	6.4 ± 0.2	9.8 ± 0.7	7.3 ± 0.6	4.8 ± 0.4	**1.53**	1.14	**0.75**

a The ratio of the metal content in wild type and mutants is shown. The values represent averages and standard deviation from three biological measurements. The bold letters indicate significant changes in the ratio column (*P* < 0.05, Student’s *t* test).

Ca and Zn levels were not drastically altered in the mutants. In contrast, Cu, which influences the carotenoid content in cyanobacteria (52), was enhanced in I-*tonB2*, in which the carotenoid level was found to be enhanced as well (Tables 1 and 2). In turn, in I-*tonB3* and I-*tonB4* the Co levels were reduced, which in the case of I-*tonB4* could be related to the reduced carotenoid level (Tables 1 and 2). In summary all *tonB* mutants exhibited alterations in the cellular metal levels compared to wild type to different extents. Whereas I-*tonB3* and I-*tonB4* were reduced in Co, Mn (I-*tonB3*), or Mo (I-*tonB4*), only I-*tonB2* did, besides the observed reduction in Mn, significantly enrich metals, namely, Cu and Mo.

### Membrane properties and transcriptional alterations in I-*tonB2*.

**FIG 3:**
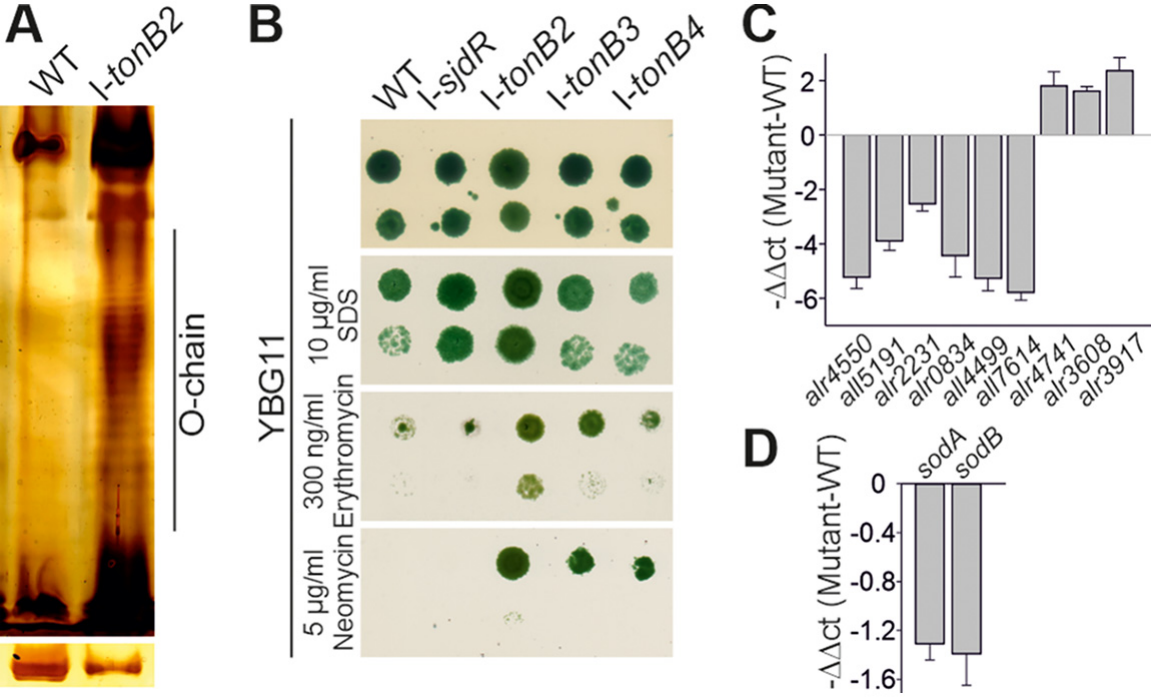
Outer membrane integrity of the *tonB* mutants and expression patterns in I-*tonB2*. (A) LPS extracted from the wild type (WT) and I-*tonB2* was separated by SDS-PAGE and silver stained. The O-antigen fragments are indicated. The loading control (large subunit of Rubisco) is given in the bottom panel where whole-cell lysate of both strains was separated in amounts proportional to the LPS extract. A representative result is shown; other LPS analyses performed from independently grown cultures are shown in Fig. S2. (B) The wild type (WT) and the *tonB* mutants were spotted on plates of YBG11 medium supplemented with the indicated compounds. Five microliters of a cell suspension with an OD_750_ of 1 (upper row in each plate) or an OD_750_ of 0.1 (bottom row in each plate) was spotted. Images were taken after 10 days of incubation. The experiment was conducted three times with independent cultures, and representative images are shown here. (C and D) The relative expression (I-t*onB2* versus WT) in terms of −ΔΔ*C_T_* is shown for the putative *Anabaena* porin genes (C) and the superoxide dismutase genes (D). The Δ*C_T_* value was normalized to the housekeeping gene *rnpB* (giving the Δ*C_T_*) and the respective Δ*C_T_* of the wild type (giving the ΔΔ*C_T_*). Average values from three independent biological replicates are shown; error bars indicate the standard deviation.

Considering the relative accumulation of Cu and Mo in I-*tonB2* and the tendency of this strain to form aggregates in solution, a modification in the cell surface could cause the mentioned effects. Therefore, lipopolysaccharide (LPS) was extracted from the wild-type and I-*tonB2* strains and separated by SDS-PAGE. Reproducibly enhanced signals for the O-antigen ladder were observed for the *tonB2* mutant compared to wild type, in which the O-chain was barely visible when similar amounts of LPS extracts were loaded (Fig. 3A and Fig. S2). This confirms an increased synthesis of LPS in the mutant strain that could result from an aberrant regulation.

10.1128/mSphere.00214-21.2 FIG S2 LPS extractions from wild-type *Anabaena* and I-*tonB2* cultures. (1) Cultures grown in tubes in the presence of 1% CO_2_. (2) Cultures grown without bubbling in shaking flasks; a higher degree of aggregation was observed without CO_2_ supplementation. The LPS isolated from the cultures was separated by SDS-PAGE and silver stained. The bottom panel shows the loading control (large subunit of Rubisco). Download FIG S2, PDF file, 1.6 MB. Copyright © 2021 Schätzle et al. 2021 Schätzle et al. https://creativecommons.org/licenses/by/4.0/ This content is distributed under the terms of the Creative Commons Attribution 4.0 International license.

LPS structure as well as carotenoids are known to influence membrane properties (55–59). In cyanobacteria, carotenoids are found in all membranes including the outer membrane (60–62). Therefore, the integrity of the OM in the *tonB* mutants was tested by spotting suspensions of the strains on plates containing antibiotics or SDS. Here, I-*sjdR* was utilized as an unrelated control strain bearing a plasmid insertion.

Interestingly, I-*tonB2* exhibited an increased resistance toward SDS, erythromycin, and neomycin compared to the wild type (Fig. 3A). In addition, I-*tonB3* and I-*tonB4* were more resistant toward the selected antibiotics, but the effect was not as pronounced as for I-*tonB2*. Moreover, I-*sjdR* grew in a similar manner as I-*tonB2* in the presence of SDS but was as sensitive toward the tested antibiotics as the wild type.

The reduced susceptibility of I-*tonB2* suggests a limited uptake of the selected compounds into the cell and confirms an alteration to the cell envelope. Typically, porins mediate the transport of certain antibiotics across the OM, and thus, porin mutants often display hyperresistance toward those compounds (63). Although it has been discussed that lipophilic macrolide antibiotics likely enter the cell through diffusion across the membranes and not through porins (17), for *Anabaena* a relation to porin function has been proposed (64). Thus, the transcript abundance of nine genes coding for porin-like proteins (1, 65) was examined in I-*tonB2*.

Remarkably, the transcript abundance of six genes coding for putative porins was reduced in I-*tonB2* compared to the wild type after 7 days of growth in YBG11 medium (Fig. 3B). Among those, the transcript abundance of *all7614*, *all4499*, and *alr4550* was most drastically reduced by 54-, 37-, and 36-fold, respectively. The transcript level of the three genes *alr4741*, *alr3608*, and *alr3917* was higher in the mutant than in the wild type. The maximum increase was, however, 5-fold (*alr3917*), which appears to be only moderate compared to the drastic downregulation of other putative porin-encoding genes (Fig. 3B).

In addition to their impact on membranes, carotenoids are involved in the protection of the photosynthetic apparatus from reactive oxygen species (ROS). Thus, an increased carotenoid concentration might result from an elevated level of oxidative stress. To test whether I-*tonB2* exhibits a higher oxidative stress level, the expression of superoxide dismutase A (SodA, MnSOD) and B (SodB, FeSOD) was analyzed. Both enzymes confer resistance to oxidative stress under distinct nitrogen regimes (66). The abundance of both transcripts was reduced in I-*tonB2* in comparison to the wild type after 7 days of cultivation in YBG11 medium (Fig. 3C). This suggests that the *tonB2* mutant does not suffer from increased oxidative stress. The assessment of reactive oxygen (ROS) production in I-*tonB2* and wild type by the fluorescent probe 2,7-dichlorodihydrofluorescein diacetate (DCFH-DA) confirmed this finding, as no difference in fluorescence that represents cellular ROS content was detected (Fig. S3).

10.1128/mSphere.00214-21.3 FIG S3 Estimation of intracellular ROS level. Cultures of wild type and I-*tonB2* were grown for 1 week to a density of ∼OD_750_ of 1, and intracellular ROS levels were determined using the DCHF-AC assay (112) after 20-min pretreatment in the dark, ambient light, or UV-B radiation with or without addition of ascorbic acid. Higher conversion to fluorescent dichlorofluorescein indicates higher ROS levels. I-*tonB2* did not behave significantly different from the wild type (Student’s *t* test). Download FIG S3, PDF file, 0.2 MB. Copyright © 2021 Schätzle et al. 2021 Schätzle et al. https://creativecommons.org/licenses/by/4.0/ This content is distributed under the terms of the Creative Commons Attribution 4.0 International license.

### I-*tonB2* and I-*tonB4* are impaired in nitrogenase activity.

**FIG 4:**
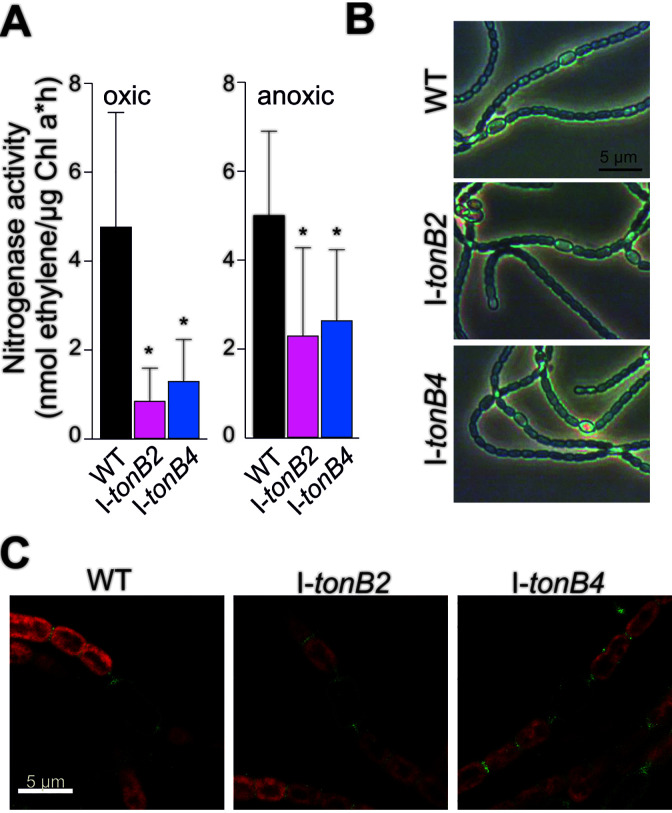
Heterocyst formation and nitrogenase activity in I-*tonB*2 and I-*tonB4*. (A) Nitrogenase activity was determined under oxic and anoxic conditions in the wild type (WT), I-*tonB2*, and I-*tonB4*. Average values of 9 (wild type), 4 (I-*tonB2*), and 5 (I-*tonB4*) measurements on independent cultures are given, and error bars represent the standard deviation. Asterisks denote significant differences between each mutant and the wild type (Student’s *t* test, *P* < 0.05). (B) Light microscopy images of cultures grown for 3 days on BG11_0_ medium plates; representative images are shown. (C) The peptidoglycan of cells grown for 7 days on BG11_0_ medium plates was labeled with the fluorescent dye Van-FL that specifically binds to the cell wall (see Materials and Methods for details). Fluorescence images were recoded with a confocal laser scanning microscope; a merge of the Van-FL fluorescence (green) and the chlorophyll autofluorescence (red) is shown. For better visibility, the Van-FL signal and the overall intensity were enhanced.

The nitrogenase enzyme in *Anabaena* has Mo as a cofactor (12). Notably, I-*tonB2* and I-*tonB4* were altered in the cellular Mo concentration compared to wild type (Table 2), and the abundance of the *tonB4* transcript was found to be enhanced in the absence of combined nitrogen (38). Therefore, the nitrogenase activity was determined for I-*tonB2* and I-*tonB4* by means of the acetylene reduction assay. Under oxic conditions, a nitrogenase activity of 0.8 ± 0.7 or 1.3 ± 0.9 nmol ethylene/μg Chl · h was measured in I-*tonB2* and I-*tonB4*, respectively (Fig. 4A), which was significantly lower than in the wild type (4.7 ± 2.6 nmol ethylene/μg Chl · h). Under anoxic conditions, the nitrogenase activity in both mutants was enhanced compared the oxic conditions. However, the average values of 2 ± 2 and 3 ± 2 nmol ethylene/μg Chl · h for I-*tonB2* and I-*tonB4*, respectively, were again significantly lower than the nitrogenase activity of the wild type. Hence, both strains showed a reduced but not abolished nitrogen fixation capacity.

Considering the alterations in nitrogenase activity, the heterocysts of the *tonB* mutants were analyzed under the light microscope and compared to those of the wild type. I-*tonB2* and I-*tonB4* mutants differentiated wild-type-like heterocysts as judged from light microscopy (Fig. 4B), and the formation of the constricted heterocyst pole was not altered as determined with fluorescently labeled vancomycin (Fig. 4C). Therefore, TonB2 and TonB4 are not essential for heterocyst differentiation, although *tonB4* expression is upregulated under nitrogen starvation (38) and nitrogenase activity in the mutants is somewhat reduced compared to the wild type (Fig. 4A).

### Iron starvation and siderophore transport capacities of *tonB* mutants.

Next, we addressed the question whether TonB2 and TonB4 are involved in the transport of ferric siderophores, as described for TonB3 (39). First, a potential complementation of single *tonB* insertions through enhanced expression of other *tonB* genes was analyzed by quantitative reverse transcription-PCR (qRT-PCR). SjdR was excluded from this analysis, as it is no longer considered a TonB candidate (39). The transcript abundance of the nonmutated *tonB* genes was determined after 7 days of iron starvation and normalized against the expression of the housekeeping gene *rnpB*. Starvation was applied since the expression of genes involved in TonB-dependent transport in *Anabaena* is triggered in the absence of iron (38, 42, 48, 67).

**FIG 5:**
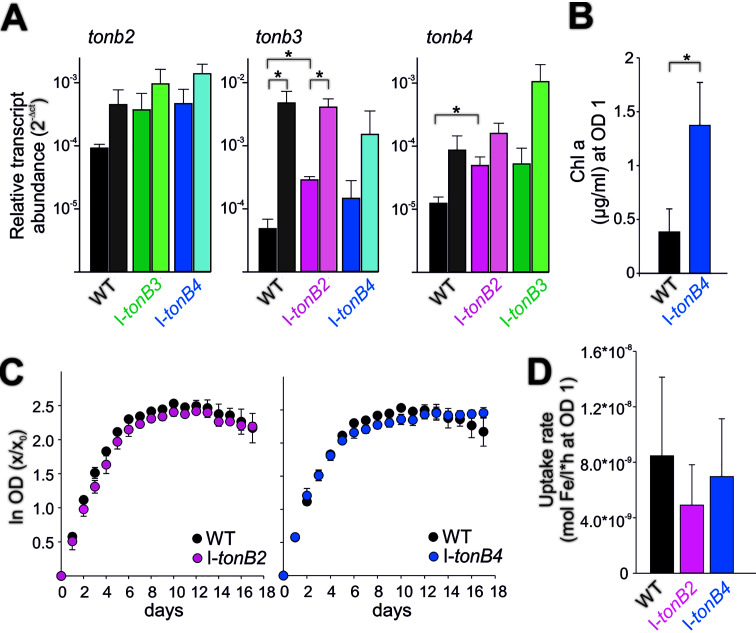
Gene expression, growth, and siderophore transport in the *tonB* mutants. (A) RNA isolated from wild-type and *tonB* mutant strains after growth in YBG11 medium (left bars in black, dark green, dark blue, and dark pink) and after 7 days of iron starvation (right bars in gray, light green, light blue, and light rose). Gene expression was normalized to *rnpB*. Significant changes between the mutants and the wild type or the two conditions tested in one strain are marked with an asterisk (Student’s *t* test, *P* < 0.05). Three independent biological replicates per strain were analyzed, and the error bars represent the standard deviation. (B) Cultures were grown for 25 days in iron-free medium, and Chl was extracted; averages from three different biological replicates per strain with standard deviation are shown. Asterisk indicates *P* < 0.05 (Student’s *t* test). (C) Wild-type, I-*tonB2*, and I-*tonB4* cultures were prestarved and diluted afterward to an OD_750_ of 0.05, and growth was monitored. The growth of three different cultures per strain was analyzed; for better visualization and comparison, the wild-type values are present in both diagrams. Standard deviations are indicated. (D) Ferric schizokinen transport was measured with the wild type, I-*tonB2*, and I-*tonB4*. Average values of 8 (wild type), 7 (I-*tonB2*), and 7 (I-*tonB4*) measurements from independent cultures are presented, and standard deviations are given.

The abundance of *tonB2* transcripts in I-*tonB3* and I-*tonB4* mutants was not significantly different from the wild-type level, both under standard conditions and after iron starvation (Fig. 5A, left). Further, no significant alteration of *tonB2* expression was observed in response to iron starvation in any of the strains tested. Similarly, the expression of *tonB4* was not significantly affected by iron starvation (Fig. 5A, right) but was enhanced in I-*tonB2* compared to the wild type when strains were grown in YBG11 medium.

The *tonB3* transcript was increased under iron starvation in all strains (Fig. 5A, middle), although the change in I-*tonB4* was not significant (Fig. 5A, middle). Moreover, *tonB3* expression in I-*tonB2* was enhanced compared to the wild type when cells were grown in YBG11 medium but not after iron starvation. This may suggest an early level of starvation in I-*tonB2*, since *tonB3* expression is triggered upon iron depletion. However, no changes in Chl (as indicator of iron starvation) and no significant alteration in the cellular iron content were observed in I-*tonB2* compared to the wild type (Tables 1 and 2), which does not support iron starvation in I-*tonB2*.

In contrast, *tonB3* expression was not as drastically enhanced after iron starvation in I-*tonB4* as observed in the wild type or I-*tonB2*. This suggests a comparatively lower level of starvation in the *tonB4* mutant. To test this, wild-type and mutant cultures were grown for 25 days in iron-free medium and the Chl level was determined as an indicator for the degree of starvation. Indeed, the Chl content was more drastically decreased in the wild type (0.38 μg ml^−1^ at an OD_750_ of 1) than in I-*tonB4* (1.4 μg ml^−1^ at an OD_750_ of 1) (Fig. 5B). Thus, the *tonB4* mutant shows an unusually weak iron starvation phenotype, which explains the reduced induction of *tonB3* expression during starvation.

In conclusion, no drastic alterations of *tonB* transcript abundance were observed between the mutants and the wild type after iron starvation. Thus, complementation effects between the *tonB* genes appear not to take place according to the analysis of the phenotypes under iron starvation. Then, the growth behavior in iron-free medium was analyzed for I-*tonB2* and I-*tonB4* (Fig. 5C). The cultures were prestarved prior to monitoring growth, since iron starvation in *Anabaena* which involves the expression of relevant uptake systems requires initiation time (67). We did not observe a compromised growth of the mutants compared to the wild type, which argues against a direct function of TonB2 and TonB4 in iron uptake. To support this conclusion, the transport rates of schizokinen loaded with ^55^Fe were determined. Although the normalized uptake rates of 4.9 × 10^−9^ mol Fe/liter · h for I-*tonB2* and 7.0 × 10^−9^ mol Fe/liter · h for I-*tonB4* were slightly lower than the wild-type rates (8.5 × 10^−9^ mol Fe/liter · h), no significant difference could be established (Fig. 5D). Thus, TonB2 and TonB4 do not seem to function in schizokinen transport in *Anabaena*.

## DISCUSSION

The TonB-ExbB-ExbD system is conserved among Gram-negative bacteria, as approximately two-thirds of these bacteria have at least one TonB-encoding gene (3). Notably, many species encode multiple TonB copies in the genome (3), and diverse functions have been assigned to the different genes in one species (30, 31, 36).

In *Anabaena* four genes have been annotated as encoding possible TonB proteins. For SjdR (formerly annotated as TonB1), a function distinct from TonB-dependent transport was described (39). With respect to the three TonB-like proteins of *Anabaena* that exhibit a conserved domain architecture (Fig. 1), TonB3 represents the central component of the siderophore-dependent iron uptake system (38, 39). In contrast, TonB2 and TonB4 are likely not related to iron uptake, since schizokinen uptake is not impaired in their mutants (Fig. 5). The abnormal iron starvation behavior of I-*tonB4* requires further investigation and cannot be explained at this stage. Notably, *Anabaena* is capable of transporting other siderophores besides the endogenously synthesized schizokinen, such as aerobactin, ferrioxamine B (48, 67, 68), or ferrichrome (unpublished data). Aerobactin penetrates through the same TBDT as schizokinen does (67); therefore, it is likely that aerobactin transport is TonB3 dependent as well (38). That TonB2 or TonB4 is involved in the TonB-dependent transport of other iron-containing substrates cannot be excluded. However, because the growth of the mutant cultures is not affected in the absence of iron, a relation to ferric siderophore transport seems unlikely. Besides ferric siderophores, also other substrates such as cobalamin, nickel, or sugars are transported in a TonB-dependent manner in some organisms (16, 19). This further broadens the spectrum of possible functions for TonB2 and TonB4 that will need to be investigated, especially considering the high number of 22 TBDTs that are predicted from the *Anabaena* genome (37).

The phenotypes of I-*tonB2* and I-*tonB4* were investigated in order to figure out possible functions. Differential characteristics of the strains were unveiled, including alterations of the cellular metal concentrations that were observed, albeit to different extents, for all *tonB* mutants. Both I-*tonB3* and I-*tonB4* show decreased cellular contents of Mg and Co, and I-*tonB4* also shows a decreased content of Mo (Table 2). Molybdenum is the cofactor of the nitrogenase enzyme (12), which might contribute to the reduced nitrogenase activity in I-*tonB4* (Fig. 4). Moreover, in I-*tonB4* the Chl level is comparatively decreased (Table 1), which in turn might be a consequence of the reduced Mg content in this strain. Notably, in I-*tonB4* the regulation of Chl synthesis seems to be generally affected, considering the remarkably high Chl concentration after iron starvation.

Besides a decrease in Mn in I-*tonB2* and I-*tonB3*, an accumulation of Cu and Mo was observed in I-*tonB2* that might be caused by altered outer membrane properties. The anionic LPS surface is involved in metal binding in bacteria (69–72), and it was reported previously that cyanobacterial negatively charged exopolysaccharides are capable of binding metals (73) and might even accumulate trace metals under starvation conditions. Thus, the enhanced LPS production in I-*tonB2* might (i) lead to an enhanced adsorption of certain metals that subsequently diffuse into the cell and (ii) cause the formation cell aggregates.

LPS is thought to have an important role in porin trimerization, stability, and conductance (74–76). In the so-called deep rough mutants, strains that are compromised in LPS synthesis and thus produce only truncated LPS, a smaller amount of protein is present in the OM (17). Additionally, those mutants are increasingly susceptible to SDS or hydrophobic antibiotics (77, 78). An opposite effect might take place in I-*tonB2*, in which the excessive LPS production might result in the monitored decrease in susceptibility toward drugs, possibly reinforced by the reduction of the expression of genes encoding porins observed in this strain. Although it has been speculated that aminoglucoside antibiotics cross the OM through a diffusion-based self-promoted pathway in which the LPS surface is involved (17, 79), for *Anabaena* a relation of erythromycin uptake to porin function has been established (64). In the I-*tonB2* mutant most of the genes coding for porins are downregulated, among them the porins described to be most abundant in *Anabaena* (40, 65). The decreased porin expression could display a feedback transcriptional response to a putatively enhanced substrate (metal) diffusion into the cell, reflected by a higher metal adsorption in I-*tonB2.* Hence, considering these characteristics a TolA-like function could be proposed for TonB2 rather than a TonB-like function.

The TolA-TolQ-TolR system embedded in the PM (Tol system here) (80) is structurally related to the TonB system. The C-terminal domains of TonB and TolA are structurally analogous (81), and it is assumed that the Tol system is involved in maintaining OM integrity (82–84). TolA interacts with trimeric porins of E. coli (85, 86), and the Tol-Pal system constitutes a component of the divisome involved in cell constriction and peptidoglycan remodeling (87, 88). Moreover, TolA might modulate the expression of LPS components in E. coli (89, 90), which also might be the case for TonB2.

The lack or low dosage of any TonB-like protein induces pigment alterations in *Anabaena* (Table 1). The reduction of the phycocyanin level in I-*tonB2* is also reflected in the comparatively bright color of its cultures (Fig. 2). Phycocyanin-containing cyanobilins are accessory pigments that harvest light and transfer energy to photosystem II. A possible explanation for the modifications in pigment content is the observed alterations in cellular metal contents because different metal treatments are known to affect cyanobacterial pigment concentrations. For example, the treatment of *Anabaena oryzae* with 1 to 100 ppm of Cu resulted in an increased carotenoid concentration after 6 to 8 days of incubation (52).

In summary, the four TonB-like proteins found in *Anabaena* apparently take over distinct functions. Neither I-*toB2* nor I-*tonB4* is drastically reduced in schizokinen uptake capacity, which suggests that TonB3 exclusively mediates schizokinen transport in *Anabaena*. On the other hand, both *tonB2* and *tonB4* mutants are compromised in the production of full nitrogenase activity, especially under oxic conditions, although heterocyst differentiation seems not affected. TonB2 influences OM properties, including LPS synthesis, with an effect on susceptibility toward antibiotics and porin abundance (as deduced from porin gene expression), and its role might be related to that of the Tol system as discussed above. The role of TonB4 is elusive, consistent with its peculiar predicted structure (Fig. 1), although we note that its mutant is affected in the cellular levels of several metals, including Mo, that may result in the observed low nitrogenase levels.

## MATERIALS AND METHODS

### *Anabaena* culture conditions.

*Anabaena* (also known as *Nostoc*) sp. strain PCC 7120 was stored on plates of BG11 medium with 1% (wt/vol) Bacto agar (BD Biosciences) (91). For liquid culturing either BG11 (91) or YBG11 medium (92) was used. *Anabaena* cultures were grown under constant shaking at 90 to 100 rpm and constant illumination (ambient light, 70 μmol photons m^−2^ s^−1^; Osram L 58 W/954-Lumilux de Luxe, daylight) at 28°C. In the case of mutant strains 5 μg ml^−1^ of both spectinomycin dihydrochloride pentahydrate (Sp; Duchefa Biochemie) and streptomycin sulfate (Sm; Roth) was added. The growth was monitored spectrophotometrically in terms of optical density at 750 nm (OD_750_). For growth analysis on plates, 5 μl of cell suspensions with an OD_750_ of 1 was spotted in a dilution series (1, 1:10, and 1:100), and representative results are shown.

### DNA extraction, molecular cloning, and generation of *Anabaena* mutants.

Transformation of E. coli and isolation and manipulation of plasmid DNA were performed according to standard protocols (93). *Anabaena* genomic DNA (gDNA) was isolated as described previously (94) with modifications: sodium dodecyl sulfate was not added, and the phenol extraction was done once followed by two washing steps with 400 μl of chloroform.

The *Anabaena tonB* mutants AFS-I-*sjdR*, AFS-I-*tonB2*, AFS-I-*tonB3*, and AFS-I-*tonB4* were utilized in this study and have been introduced previously (38, 39). The annotation stands for *Anabaena* mutant generated in Frankfurt, Germany by the Schleiff Lab by plasmid insertion; for better readability “AFS-” is omitted throughout the text. In brief, internal fragments of the single genes were ligated into vector pCSV3 (95, 96) in the case of I-*tonB2*, I-*tonB3*, and I-*tonB4* or pCSEL24 (97) in the case of I-*sjdR*, both carrying spectinomycin and streptomycin resistance markers. The oligonucleotides and the plasmids used in this study are listed in Tables S1 and S2 in the supplemental material, respectively. Plasmids were transferred to *Anabaena* with the triparental mating method as previously described (40, 97–99). The *Anabaena* strains analyzed in this study are listed in Table S3. The genotype of the exconjugants was tested by PCR, in which an oligonucleotide specific for the plasmid in combination with an oligonucleotide specific for the gene region was used.

10.1128/mSphere.00214-21.4 TABLE S1 Oligodeoxynucleotides used in the study. Download Table S1, DOCX file, 0.01 MB. Copyright © 2021 Schätzle et al. 2021 Schätzle et al. https://creativecommons.org/licenses/by/4.0/ This content is distributed under the terms of the Creative Commons Attribution 4.0 International license.

10.1128/mSphere.00214-21.5 TABLE S2 Plasmids used in this study. Download Table S2, PDF file, 0.09 MB. Copyright © 2021 Schätzle et al. 2021 Schätzle et al. https://creativecommons.org/licenses/by/4.0/ This content is distributed under the terms of the Creative Commons Attribution 4.0 International license.

10.1128/mSphere.00214-21.6 TABLE S3 *Anabaena* strains used in this study. Download Table S3, PDF file, 0.09 MB. Copyright © 2021 Schätzle et al. 2021 Schätzle et al. https://creativecommons.org/licenses/by/4.0/ This content is distributed under the terms of the Creative Commons Attribution 4.0 International license.

### Short-term siderophore transport measurements.

The transport rates of ferric schizokinen were determined as described earlier (67, 100). Schizokinen was ordered from EMC Microcollections, and ^55^FeCl_3_ was purchased from Perkin-Elmer. A final concentration of 15 nM ^55^Fe-schizokinen was used in single measurements, and the final cell suspension utilized for measuring was inoculated at an OD_750_ of 0.05. Cultures were prestarved in iron-free YBG11 before the measurements were conducted, and the degree of starvation was estimated by the chlorophyll *a* (Chl) concentration at an OD_750_ of 1, as previous studies indicated that the Chl concentration per cell decreases in *Anabaena* with ongoing iron starvation (48). The Chl concentrations of experimental cultures are given in Table S4.

10.1128/mSphere.00214-21.7 TABLE S4 ^55^Fe-Schizokinen uptake rates and normalized chlorophyll values. Download Table S4, DOCX file, 0.01 MB. Copyright © 2021 Schätzle et al. 2021 Schätzle et al. https://creativecommons.org/licenses/by/4.0/ This content is distributed under the terms of the Creative Commons Attribution 4.0 International license.

### Inductively coupled plasma mass spectrometry (ICP-MS).

The cellular metal concentrations in *Anabaena* were determined as described earlier (101). In brief, cultures grown for 7 days in YBG11 were harvested by centrifugation and washed twice with 20 mM 2-(*N*-morpholino)ethanesulfonic acid (pH 5), 10 mM EDTA. Cells were resuspended in 5 ml double-distilled water, and 1 ml was subjected to inductively coupled plasma mass spectrometry (ICP-MS) measurement. The OD_750_ of the suspension was measured, and cells were counted for normalization. Samples were digested overnight at 120°C in 7 M HNO_3_ and dissolved in 5% HNO_3_ for measurement.

### Pigment extraction.

The measurements of Chl and carotenoid (Car) concentrations were performed with methanolic extracts as previously described (102, 103). The Chl concentration was determined with the formula Chl (μg/ml) = 12.9447(*A*_665_ − *A*_720_), and the carotenoid concentration was calculated with the formula Car (μg/ml) = {1,000(*A*_470_ − *A*_720_) − 2.86 × [Chl (μg/ml)]}/221.

To estimate the Chl concentration for experimental cultures, a simplified equation was utilized, as follows: Chl (mg/ml) = *A*_665_/74.5 (104).

Determination of phycocyanin content was carried out as described by Horvath et al. (105). In brief, cultures were harvested by centrifugation (1 min, 4,000 × *g*) and concentrated to a volume of 0.3 to 2 ml. OD_750_ of the concentrates was determined in 1/100 dilution, and the remaining material was frozen at −20°C. After thawing at room temperature (RT), a defined volume was diluted to 4 ml with phosphate-buffered saline (PBS) and subjected to sonication with a sonication probe at full power for 90 s. Cell debris was removed by centrifugation (1 min, 20,000 × *g*). PC concentration was calculated from UV-visible (UV-Vis) absorption spectra as described previously (106): PC (μg/ml) = 154(*A*_618_ − *A*_730_).

### Van-FL staining and microscopy.

For visualization of peptidoglycan, the filaments were stained with BODIPY FL vancomycin (Van-FL) (Invitrogen) as previously described (107). For microscopy a piece of agar was excised and reversely placed onto a coverslip that was utilized as a microscope slide. Images were recorded with a Zeiss LSM 780 using 63× or 40× objectives with immersion oil. Diameter of the pinhole was set to 69.4 μm, and a 488-nm laser source was used for excitation. Specific Van-FL fluorescence was recorded between 500 and 550 nm, Chl autofluorescence was recorded between 630 and 700 nm. Light microscopy images were recorded with a Thorlabs DCC1645C-HQ camera.

### RNA extraction, RT-PCR, and qRT-PCR.

RNA was isolated either from strains that had been grown for 7 days in YBG11 medium or, in the case of iron starvation, from samples incubated for 7 days under culture conditions in iron-free YBG11 medium. RNA was extracted with TRIzol (Thermo Fisher Scientific) according to previously described protocols (42). After DNase I digestion the absence of DNA in RNA samples was verified by PCR. Revert Aid reverse transcriptase was used for first strain cDNA synthesis (Thermo Fisher Scientific). qPCR was performed with a StepOnePlus cycler from Thermo Fisher Scientific, and the cycling conditions were set as 50°C, 2 min, and 95°C, 2 min, followed by 40 cycles at 95°C for 15 s, 60°C for 30 s, and 72°C for 30 s. Experiments were performed with PowerUp SYBR green Master Mix from Applied Biosystems. *rnpB* served as control gene. The threshold cycle (*C_T_*) value of the gene of interest was normalized to the *rnpB C_T_* value (Δ*C_T_*) and, if indicated, further normalized to the corresponding Δ*C_T_* of the wild-type (ΔΔ*C_T_*) (108).

### Extraction and analysis of lipopolysaccharide.

Lipopolysaccharide (LPS) was extracted from wild type and I-*tonB2*, and the cultures were grown in flasks with constant shaking for 8 days or in tubes with supplementation of 1% CO_2_ in YBG11 medium for 7 days (Fig. S2). Cells corresponding to an OD_750_ of 3 in 1 ml were harvested for LPS extraction. LPS was extracted as described previously (109) with modifications. In brief, the cells were harvested by centrifugation (6,000 × *g*, 5 min), washed with 1 ml of phosphate-buffered saline (137 mM NaCl, 2.7 mM KCl, 10 mM Na_2_HPO_4_, 1.8 mM KH_2_PO_4_), and resuspended in 50 μl lysing buffer (2% sodium dodecyl sulfate, 4% β-mercaptoethanol, 10% glycerol, 1 M Tris-HCl, pH 6.8, bromphenol blue). After the samples were heated at 100°C for 10 min, 10 μl of lysing buffer containing 100 μg proteinase K was added, followed by 60 min of incubation at 60°C.

The LPS was subjected to denaturing SDS-polyacrylamide electrophoresis utilizing a 12% acrylamide gel; afterward the gel was silver stained (110). For the loading control (large subunit of Rubisco is shown in figures), cells were treated as described for LPS extraction omitting proteinase K.

### Nitrogenase activity.

Nitrogenase activity was determined by the acetylene reduction assay (111) carried out under both oxic and anoxic conditions. Cells grown in 25 ml of liquid BG11 medium (supplemented with Sp/Sm when necessary) were harvested by centrifugation, washed with liquid BG11_0_ medium, and incubated at 1 μg Chl ml^−1^ (without antibiotics) in 25 ml of liquid BG11_0_ medium for 48 h. Cell suspensions of 2 ml at 6 μg Chl ml^−1^ were transferred to sealed small flasks in which the nitrogenase activity assay was carried out. Under both oxic and anoxic conditions 2 ml of acetylene was injected. For the assays under oxic conditions, the sealed flasks were incubated for 30 min (30°C and shaking) before taking 1-ml samples for determination of ethylene by gas chromatography. For anoxic conditions, the sealed flasks were supplemented with 10 μM 3-(3,4-dichlorophenyl)-1,1-imethylurea (DCMU; from Sigma), bubbled with argon for 4 min, and incubated for 60 min (30°C and shaking) before acetylene injections, and then 1-ml samples were taken for ethylene determination. Under both conditions, samples for ethylene determination were taken for up to 2 h.

### Determination of ROS production.

Intracellular conversion of dichlorodihydrofluoresein (DCHF) to dichlorofluorescein (DCF) was measured as established previously (112). Cultures were harvested by centrifugation (1 min, 4,000 × *g*) and rediluted to an OD_750_ of 1 with PBS. For comparison, ascorbic acid (100 μM final concentration) was added to some samples. Samples of 10 ml were subjected to the respective light treatment for 20 min. UV treatment was carried out by placing the samples on the transilluminator of a gel electrophoresis documentation system, and ambient light treatment was carried out under cultivation conditions. After addition of 2,7-dichlorodihydrofluorescein (final concentration, 5 μM), samples were incubated for 1 h in the dark. Finally, cells were harvested and transferred to 96-well plates, and fluorescence and OD_750_ were determined using a microplate reader.
