# Supplementary figures and images for: Functional Diversity of TonB-Like Proteins in the Heterocyst-Forming Cyanobacterium Anabaena sp. PCC 7120
Source: mSphere. 2021 Nov 17;6(6):e00214-21. doi: 10.1128/mSphere.00214-21 (PMC8597729; doi:10.1128/mSphere.00214-21)

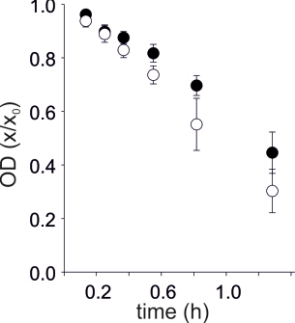

Supplement: FIG S1 [file msphere.00214-21-sf001.pdf]

WT (1)

I-tonB2 (1)

WT (2)

I-tonB2 (2)

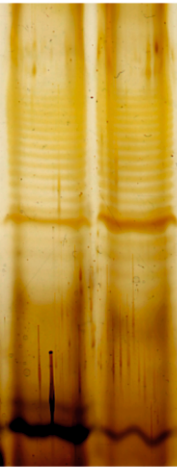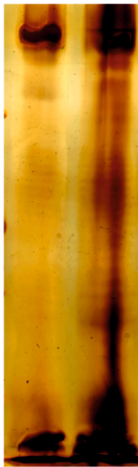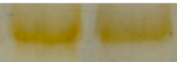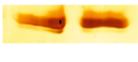

Supplement: FIG S2 [file msphere.00214-21-sf002.pdf]

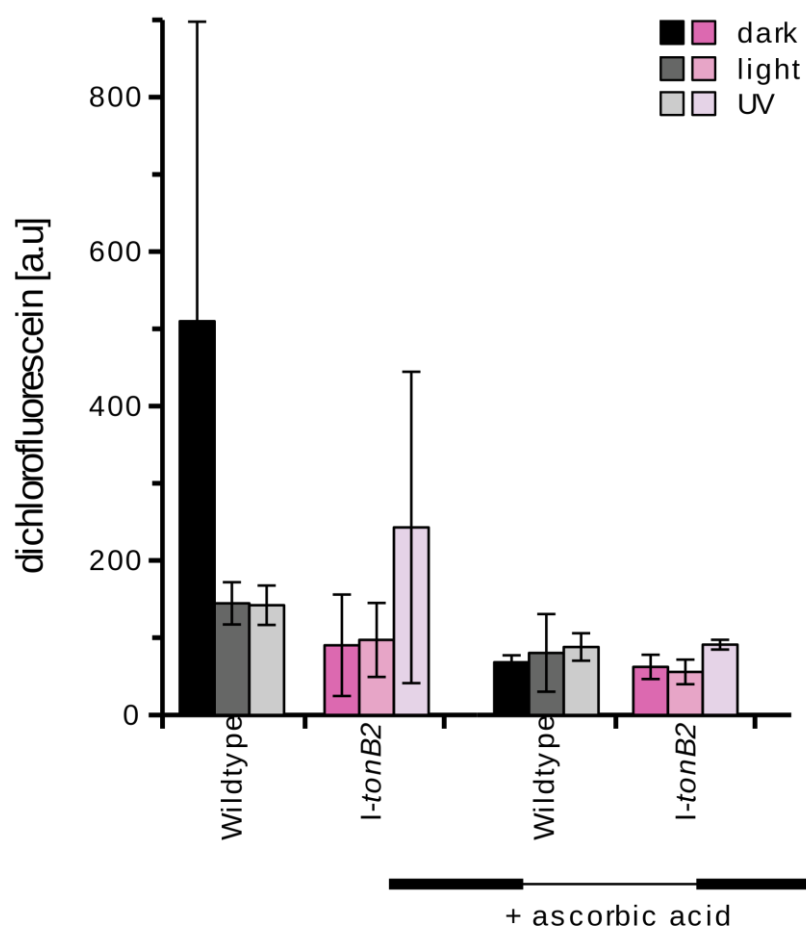

Supplement: FIG S3 [file msphere.00214-21-sf003.pdf]
