# Supplementary material for: Functional Diversity of TonB-Like Proteins in the Heterocyst-Forming Cyanobacterium Anabaena sp. PCC 7120
Source: mSphere. 2021 Nov 17;6(6):e00214-21. doi: 10.1128/mSphere.00214-21 (PMC8597729; doi:10.1128/mSphere.00214-21)
Supplement: TABLE S1 [file msphere.00214-21-st001.docx]

**Table S1. Oligodeoxynucleotides used in the study**

| Oligonucleotide | Sequence (5’-3’) | Purpose |
| --- | --- | --- |
| sjdR-fw | GAATTCGTGTTAACCGTGATG | Cloning |
| sjdR-rv | GATATCGGAGAGGGACTAGG |  |
| tonB2-fw | ATTAATAGATCTGTTGCAGTACCTCAGGGTTG |  |
| tonB2-rv | ATTAATAGATCTGATTTACCTTGCAGCGATC |  |
| tonB3-fw | ATTAATAGATCTCTGACACGAGTTCCTCAAGTTG |  |
| tonB3-rv | ATTAATAGATCTCTTCGTCTAGTTCTCGATTACCGC |  |
| tonB4-fw | AGATCTCCATTCCTTTGAATTC |  |
| tonB4-rv | AGATCTGGAACAGCCTTTGGAA |  |
| sjdR-s.fw | CCAGAATTTAACACTGGTGAG | Screening of *Anabaena* mutants |
| sjdR-s.rv | GCTCACGTCAATGCCTACC |  |
| tonB2-s.fw | GGCAATACCCACCTTACGG |  |
| tonB2-s.rv | GCGCTGTCGGACGTTATG |  |
| tonB3-s.fw | GACTAAGTTGGTGAGAATAGG |  |
| tonB3-s.rv | GGATCTGACTCTAGTTTCCC |  |
| tonB4-s.fw | GTACAATCTCAATCATTCTGG |  |
| tonB4-s.rv | CTGACGAAGTTGATCATTGC |  |
| Vector-s | CTGATGCCGCATAGTTAAGCC |  |
| rnpB-qRT.fw | GTAGGCGTTGGCGGTTG | qRT PCR |
| rnpB-qRT.rv | CACTGGACGTTATCCAGC |  |
| tonB2-qRT.fw | GAACGGGTTGCAGTACCTC |  |
| tonB2-qRT.rv | CAGCGTTTGGCGTAACAGG |  |
| tonB3-qRT.fw | GCCAGATATTCCAGCACAG |  |
| tonB3-qRT.rv | GGCAAACCTTCTGAACGAG |  |
| tonB4-qRT.fw | CCAACGCCTGTCACTATTAC |  |
| tonB4-qRT.rv | CTAACGAGACTGAAAGCACC |  |
