# Supplementary material for: Functional Diversity of TonB-Like Proteins in the Heterocyst-Forming Cyanobacterium Anabaena sp. PCC 7120
Source: mSphere. 2021 Nov 17;6(6):e00214-21. doi: 10.1128/mSphere.00214-21 (PMC8597729; doi:10.1128/mSphere.00214-21)
Supplement: TABLE S2 [file msphere.00214-21-st002.pdf]

Table S2. Plasmids used in this study

Plasmid	Resistance	Insert	Purpose	Reference
pCSV3	Sp^R/Sm^R		Cloning	Valladares, A., Rodríguez, V., Camargo, S., Martínez-Noël, G. M., Herrero, A., & Luque, I. (2011). Journal of bacteriology, 193(5), 1172–1182.
pCSEL24	Sp^R/Sm^R			Olmedo-Verd, E., Muro-Pastor, A. M., Flores, E., & Herrero, A. (2006). Journal of bacteriology, 188(18), 6694–6699.
pCSEL24-alr0248	Sp^R/Sm^R	Internal fragment of *sjdR*	Generation of single-recombinant mutants	Stevanovic, M., Hahn, A., Nicolaisen, K., Mirus, O., & Schleiff, E. (2012). Environmental Microbiology, 14(7), 1655-70.; Schätzle, H., Arévalo, S., Flores, E., & Schleiff, E. (2021). mBio, 12(3), e0048321.
pCSV3-all3585	Sp^R/Sm^R	Internal fragment of *tonB2*		
pCSV3-all5036	Sp^R/Sm^R	Internal fragment of *tonB3*		
pCSV3-alr5329	Sp^R/Sm^R	Internal fragment of *tonB4*		
