# Supplementary material for: Functional Diversity of TonB-Like Proteins in the Heterocyst-Forming Cyanobacterium Anabaena sp. PCC 7120
Source: mSphere. 2021 Nov 17;6(6):e00214-21. doi: 10.1128/mSphere.00214-21 (PMC8597729; doi:10.1128/mSphere.00214-21)
Supplement: TABLE S3 [file msphere.00214-21-st003.pdf]

Table S3. *Anabaena* strains used in this study

Strain	Resistance	Genotype	Reference
WT	-		
AFS-I-*sjdR*	Sp^R/Sm^R	*alr0248*::pCSEL24	Stevanovic, M., Hahn, A., Nicolaisen, K., Mirus, O., & Schleiff, E. (2012). Environmental Microbiology, 14(7), 1655-70.; Schätzle, H., Arévalo, S., Flores, E., & Schleiff, E. (2021). mBio, 12(3), e0048321.
AFS-I-*tonB2*	Sp^R/Sm^R	*all3585*::pCSV3	
AFS-I-*tonB3*	Sp^R/Sm^R	*all5036*::pCSV3	
AFS-I-*tonB4*	Sp^R/Sm^R	*alr5329*::pCSV3	
