# Supplementary material for: Functional Diversity of TonB-Like Proteins in the Heterocyst-Forming Cyanobacterium Anabaena sp. PCC 7120
Source: mSphere. 2021 Nov 17;6(6):e00214-21. doi: 10.1128/mSphere.00214-21 (PMC8597729; doi:10.1128/mSphere.00214-21)
Supplement: TABLE S4 [file msphere.00214-21-st004.docx]

**Table S4.** ^55^Fe-Schizokinen uptake rates and normalized chlorophyll values

| Strain | Uptake rates  (mol Fe/l*h) at OD_750_=1 | Chl / OD_750_ (mg/ml) |
| --- | --- | --- |
| Wild type | 1.6*10^-8^ | 0.0010 |
|  | 4.7*10^-9^ | 0.0011 |
|  | 9.5*10^-9^ | 0.0013 |
|  | 8.3*10^-9^ | 0.0013 |
|  | 1.8*10^-9^ | 0.0010 |
|  | 1.7*10^-8^ | 0.0010 |
|  | 6.4*10^-9^ | 0.0011 |
|  | 3.7*10^-9^ | 0.0013 |
| I-*tonB2* | 4.9*10^-9^ | 0.0009 |
|  | 9.3*10^-9^ | 0.0009 |
|  | 4.8*10^-9^ | 0.0011 |
|  | 2.2*10^-9^ | 0.0006 |
|  | 8.2*10^-9^ | 0.0010 |
|  | 3.6*10^-9^ | 0.0006 |
|  | 1.4*10^-9^ | 0.0006 |
| I-*tonB4* | 1.6*10^-8^ | 0.0008 |
|  | 4.8*10^-9^ | 0.0008 |
|  | 3.7*10^-9^ | 0.0006 |
|  | 3.7*10^-9^ | 0.0007 |
|  | 8.0*10^-9^ | 0.0009 |
|  | 7.3*10^-9^ | 0.0012 |
|  | 5.6*10^-9^ | 0.0012 |
